# A full-length enriched cDNA library and expressed sequence tag analysis of the parasitic weed, *Striga hermonthica*

**DOI:** 10.1186/1471-2229-10-55

**Published:** 2010-03-30

**Authors:** Satoko Yoshida, Juliane K Ishida, Nasrein M Kamal, Abdelbagi M Ali, Shigetou Namba, Ken Shirasu

**Affiliations:** 1 Plant Science Center, RIKEN, 1-7-22 Suehiro-cho, Tsurumi-ku, Yokohama 230-0045, Japan; 2 Department of Agricultural and Environmental Biology, Graduate School of Agricultural and Life Sciences, The University of Tokyo, 1-1-1 Yayoi, Bunkyo-ku, Tokyo 113-8657, Japan; 3 Biotechnology Laboratory, Agricultural Research Corporation, Wad Medani 126, Sudan

## Abstract

**Background:**

The obligate parasitic plant witchweed (*Striga hermonthica*) infects major cereal crops such as sorghum, maize, and millet, and is the most devastating weed pest in Africa. An understanding of the nature of its parasitism would contribute to the development of more sophisticated management methods. However, the molecular and genomic resources currently available for the study of *S. hermonthica *are limited.

**Results:**

We constructed a full-length enriched cDNA library of *S. hermonthica*, sequenced 37,710 clones from the library, and obtained 67,814 expressed sequence tag (EST) sequences. The ESTs were assembled into 17,317 unigenes that included 10,319 contigs and 6,818 singletons. The *S. hermonthica *unigene dataset was subjected to a comparative analysis with other plant genomes or ESTs. Approximately 80% of the unigenes have homologs in other dicotyledonous plants including *Arabidopsis*, poplar, and grape. We found that 589 unigenes are conserved in the hemiparasitic *Triphysaria *species but not in other plant species. These are good candidates for genes specifically involved in plant parasitism. Furthermore, we found 1,445 putative simple sequence repeats (SSRs) in the *S. hermonthica *unigene dataset. We tested 64 pairs of PCR primers flanking the SSRs to develop genetic markers for the detection of polymorphisms. Most primer sets amplified polymorphicbands from individual plants collected at a single location, indicating high genetic diversity in *S. hermonthica*. We selected 10 primer pairs to analyze *S. hermonthica *harvested in the field from different host species and geographic locations. A clustering analysis suggests that genetic distances are not correlated with host specificity.

**Conclusions:**

Our data provide the first extensive set of molecular resources for studying *S. hermonthica*, and include EST sequences, a comparative analysis with other plant genomes, and useful genetic markers. All the data are stored in a web-based database and freely available. These resources will be useful for genome annotation, gene discovery, functional analysis, molecular breeding, epidemiological studies, and studies of plant evolution.

## Background

*Striga hermonthica *is an obligate root parasite belonging to the family Orobanchaceae, and is a major constraint of crop production in sub-Saharan Africa. *S. hermonthica *infests economically important crops such as sorghum, maize, millet, and upland rice, and the yield losses caused by this species have been estimated to cost as much as US$ 7 billion annually [1]. However, methods for controlling *S. hermonthica *are not well established. Despite its agricultural importance, the molecular mechanisms controlling the establishment of parasitism are poorly understood.

The *S. hermonthica *life cycle is unique and well adapted to its parasitic lifestyle. The seeds need to be exposed to germination stimulants exudated from the host roots, such as strigolactones and ethylene; otherwise they can remain dormant in the soil for several decades [2]. The seeds are tiny and possess limited amounts of nutrients, and this restricts their growth without a host connection. When a potential host is recognized through the sensing of strigolactones or other germination stimulants, the seeds that are close to the host roots (within 5 mm) can germinate. The germinated seedlings form haustoria, which are round shaped organs specialized in host attachment and penetration [3]. The formation of haustoria also requires host-derived signal compounds. The haustoria penetrate the host roots and finally connect with the vasculature to rob the host plant of water and nutrients. This dramatic developmental transition from an autotrophic to a heterotrophic lifestyle occurs within several days.

Intensive efforts in the scientific community, mainly in the United States during the 1960s, lead to the identification of some germination stimulants. This was followed by the development of a "suicidal germination" strategy to eradicate *Striga *weeds [4]. By this strategy, a germination stimulant (in this case ethylene) is mixed in the soil to trigger germination in the absence of the hosts. This approach was used successfully to eradicate *Striga asiatica *infestations in North Carolina. Although suicidal germination was effective for controlling *S. asiatica*, this approach was not applicable for African farmers due to the high cost of the strategy and the much larger scale of infestation.

Whole genome sequencing is a valuable approach to understanding an organism. The genome sequences of growing numbers of model and crop plant species have been published in recent years, providing new insights in plant biology. The development of new generation sequencing technologies has dramatically accelerated the speed of large-scale sequencing. However, the *de novo *sequencing of the whole genome of a non-model plant is still a challenging and laborious task [5]. Expressed sequence tags (ESTs) are a less expensive alternative for gaining information about the expressed genes of an organism [6]. In particular, the ESTs from a full-length enriched cDNA library provide the complete sequences of functional proteins [7].

This study aims to provide genome scale molecular resources for understanding the parasitic processes of the obligate parasite, *S. hermonthica*. We constructed a full-length enriched cDNA library from *S. hermonthica *and generated a large-scale EST dataset by reading the sequences of individual clones from both ends. The only other genus from the family Orobanchaceae with publically available EST data is *Triphysaria *[8]. *Triphysaria *spp. are facultative hemiparasites, which are able to complete their life cycles without hosts. The comparison of our *S. hermonthica *EST dataset with those of *Triphysaria *and other non-parasitic plantspecies enabled us to identify the potentially parasite specific genes. Furthermore, our results provide the tools to analyze genetic diversity within *S. hermonthica*. We found 1,445 putative simple sequence repeats (SSRs) that could be useful as markers. We amplified the genomic regions flanking some of these SSRs from *S. hermonthica *individuals that were collected in different fields in Africa. The results revealed high sequence divergence in the *S. hermonthica *genomes. All the sequences and the annotation results are freely available on the internet [9].

## Results and Discussion

### Genome size of *S. hermonthica*

**Figure 1:**
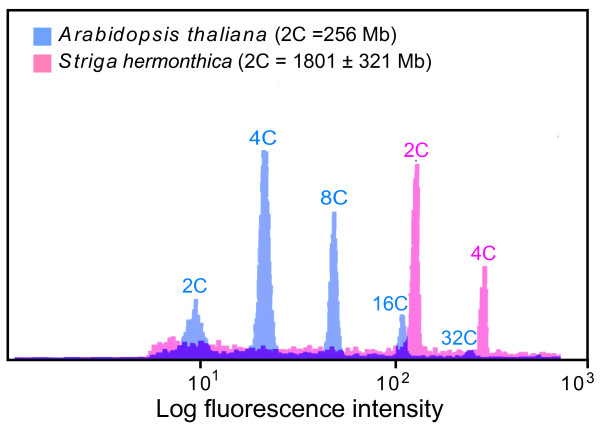
**Genome size of *S. hermonthica *estimated by flow cytometry**. The genome size of *S. hermonthica *(pink) was estimated by comparison with *Arabidopsis *(blue). n = 5.

*S. hermonthica *is likely to be a diploid species with a chromosome number of n = 19 [10]. First, we estimated the genome size of *S. hermonthica *to gain information about its genome contents. Leaves of *S. hermonthica *plants parasitizing to rice were harvested and the DNA contents were measured with a flow cytometer. *Arabidopsis thaliana*, whose genome size is 128 Mbp, was used as a control. Five individual plants were used for the measurements with two or more replicates for each plant. The genome size of *S. hermonthica *was estimated to be 1,801 Mbp (± 321 Mbp) (Fig. 1), which is approximately 14 times that of *Arabidopsis*, 4 times those of rice and poplar, and 2 times that of sorghum.

#### Full-length enriched cDNA library construction

To construct a full-length enriched cDNA library containing highly variable sequences, total RNA was extracted from various *S. hermonthica *tissues at various developmental stages (Table 1). A full-length enriched normalized cDNA library was constructed using a mixture of these RNAs as starting materials. To assess the quality of the resulting library, the inserts from 90 randomly picked clones were amplified by PCR with primers specific to the library vector, and the insert sizes were estimated by agarose-gel electrophoresis (Table 2). The average insert size was approximately 1.42 kb, which is similar to the average insert size of the RIKEN *Arabidopsis *Full-Length (RAFL) cDNA clones (estimated at 1,445 bp) [11,12]. This average insert size was similar to that of a poplar full-length cDNA library (*Populus nigra*, about 1.4 kb) [13], and slightly shorter than those from soybean and wheat (approximately 1.5 kb) [12,14]. The longest insert was estimated at more than 3 kb, suggesting that the library contains relatively long cDNAs.

**Table 1:** RNA samples used for the *S. hermonthica *full-length enriched cDNA library construction.

Tissue	Growth stage or treatment
Seedlings	At 3 d after strigol treamtment
Seedlings	At 3 d after co-incubation with rice roots
Leaves and stems	From mature plants parasitized on rice
Roots (secondary haustoria)	From mature plants parasitized on rice in rhizotron
Flowers	From mature plants parasitized on rice
Axenically grown plants	Grown axenically for 1 month

**Table 2:** Distribution of insert lengths in the *S. hermonthica *full-length enriched cDNA library.

Length (kb)	Clone number	Frequency (%)
<0.5	0	0
0.5-1.0	18	20.0
1.0-1.5	35	38.9
1.5-2.0	23	25.6
2.0-2.5	9	10.0
2.5-3.0	4	4.4
≥3.0	1	1.1
Total	90	100

*Average insert length = 1.42 kb

To assess the proportion of the library containing full-length cDNA clones, we randomly picked 90 clones and sequenced them from both the 5' and 3' ends. These DNA sequences were analyzed against the *Arabidopsis *genome database using the blastx program. Of the 90 clones, 79 contained sequences similar to those of *Arabidopsis *genes (e_value < e-10), while the insert sequences of the other 11 clones did not show any similarity. The 5'- and 3'- sequences of the 79 clones were aligned with the homologous *Arabidopsis *cDNAs. The 5'-sequences of 62 clones contained ATG start codons at similar positions to those in the corresponding *Arabidopsis *homologs, and 59 possessed stop codons at the equivalent positions. Therefore, we estimated that approximately 75% of the clones in the *S. hermonthica *library encode full-length cDNAs. Among the 59 sequenced full-length clones, the average lengths of the 5'- and 3'-untranslated regions (UTRs) were 127 bp and 203 bp, respectively, and the longest 5'-and 3' -UTRs were 486 bp and 480 bp, respectively.

### EST sequencing and statistical analysis

Next, we sequenced both the 5'- and 3'-ends of 37,710 clones from the *S. hermonthica *full-length enriched cDNA library. The sequence chromatograms were analyzed using the EST2uni package [15], which is an automated analysis tool for the clean-up, clustering, and annotation of EST sequences. Among the 75,330 raw sequence reads, we found that 67,814 were of good quality and were deposited in the DNA Databank of Japan [DDBJ: FS438984-FS506797]. The sequences are clustered into 17,137 non-redundant unigenes (10,319 contigs and 6,818 singletons) (Table 3). The average GC content among the unigene sequences is 44.5%. The lengths of the unigenes are distributed between 82 and 3,949 bp, and most of them (11,546 unigenes) have sequence lengths between 601 and 900 bp (Additional file 1), with an average of 810.3 bp. Most (84%) of the unigenes are comprised of fewer than 6 ESTs (Additional file 1), suggesting that the redundancy rate is relatively low in this normalized library.

**Table 3:** Summary of the *S. hermonthica *EST sequence analysis

Group	Records
Number of independent clones	37,710
Number of raw sequences	75,330
Number of high quality sequences	67,814
Number of unigenes	17,137
singletons	6,818
contigs	10,319
Average unigene length	775.3 bp
Minimum unigene length	101 bp
Maximum unigene length	3,051 bp
Average number of ESTs per unigene	2.9
Maximum number of ESTs per contig	106
Number of superunigenes	12,272
with more than one unigene	2,203
with one unigene	10,069
Number of putative SNPs (pSNPs)	9,299
Number of putative SSRs (pSSRs)	1,445

### Functional annotation of the unigene sequences

**Figure 2:**
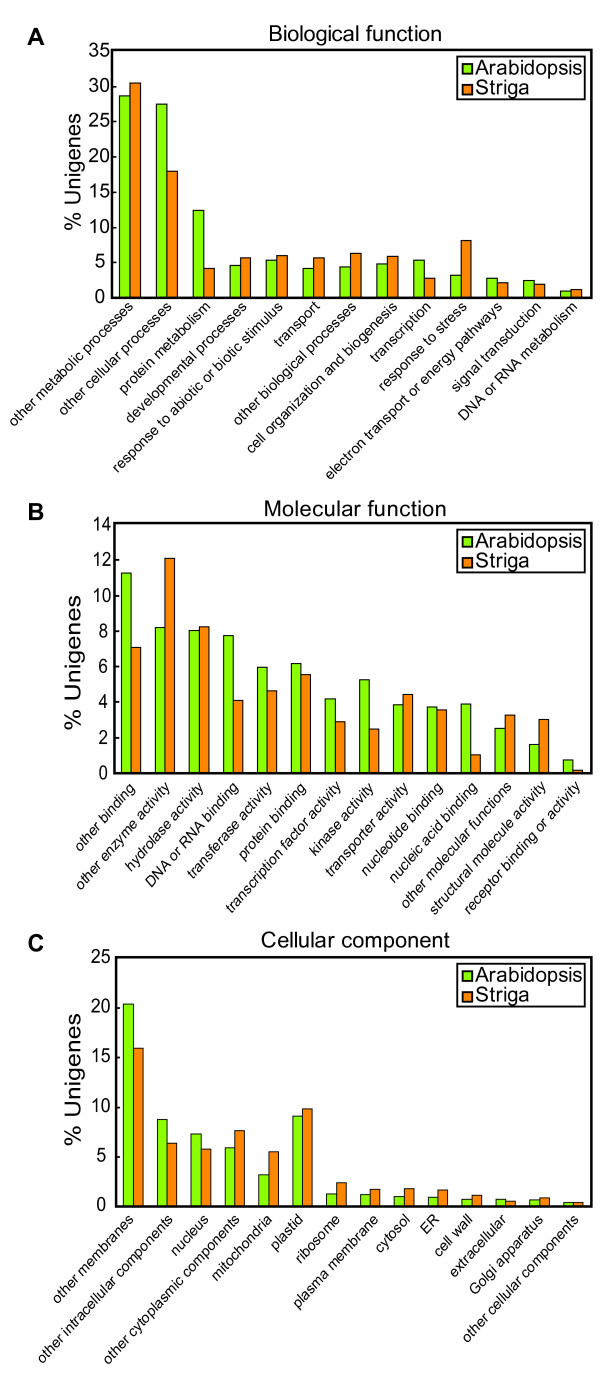
**Gene ontology analysis of *S. hermonthica *unigene-encoding products**. The *S. hermonthica *unigenes were classified according to their predicted biological functions (A), molecular functions (B), and cellular components (C). The numbers in each category were compared with those in *A. thaliana*.

For the functional annotation of the 17,137 unigene sequences, we carried out a blastx analysis against the UniRef90 database [16,17]. About 79% of the *S. hermonthica *unigenes were annotated as homologs of known proteins. For further functional annotations of the structural domains, the Pfam database [18] was searched using the HMMER program (ver. 2.3.2, [19,20]), and 31% (5367) of the unigenes contained Pfam hits. Then the *S. hermonthica *unigenes were classified into Gene ontology (GO) groups based on their similarities with the corresponding *Arabidopsis *genes (Fig. 2). In the classification of genes according to their cellular components, we found that 16% of the unigenes encode putative membrane proteins and 10% encode putative plastid proteins. In the classification of molecular functions, 12% were assigned to catalytic activity. These percentages are similar to those in *Arabidopsis *[21], indicating that there was no functional bias among the predicted proteins encoded in the *S. hermonthica *library.

### Comparative analysis with other plant genes

The *S. hermonthica *unigenes were compared with genes in other plant genomes, including *A. thaliana*, poplar (*Populus trichocarpa*), grape (*Vitis vinifera*), soybean (*Glycine max*), rice (*Oryza sativa*), sorghum (*Sorghum bicolor*), a moss (*Physcomitrella patens*), and an algae (*Chlamydomonas reindardtii*) [22-26]. Seventy-seven to seventy-nine percent of the *S. hermonthica *unigenes showed similarities with genes from other dicotyledonous plants (*Arabidopsis*, grape, soybean, and poplar), as detected by blastx (e_value < e-10). Approximately 75% of the unigenes have homologs in monocotyledonous plants (rice and sorghum), and approximately 65% and 38% showed blastx hits in the *P. patens *and *C. reindardtii *databases, respectively. These lower percentages of blast hits are consistent with the greater evolutionary distances from those organisms.

**Figure 3:**
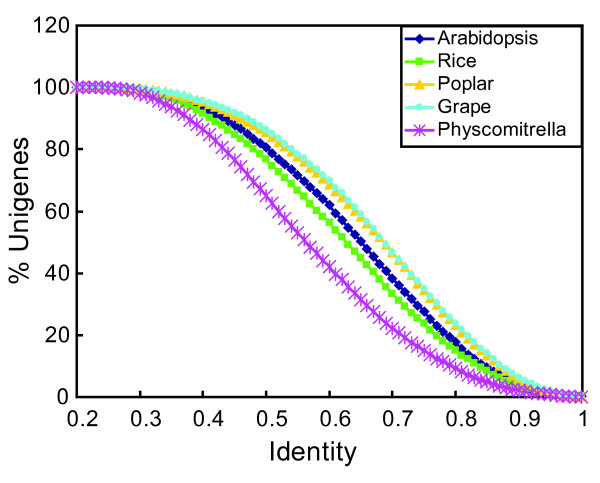
**Cumulative count curves of identity between *S. hermonthica *unigenes and those from other plant species**. All the sequenced *S. hermonthica *unigenes were used in blastx or tblastx searches against the peptide databases of the indicated plant species. The curves represent the percentages of *S. hermonthica *unigenes that showed higher levels of identity than the values on the *x*-axis.

We plotted the percentages of *S. hermonthica *unigenes against levels of amino acid sequence identity with homologs in the other plant genomes (Fig. 3). Larger percentages of *S. hermonthica *unigenes showed higher levels of identity with poplar and grape sequences than with sequences from the other plant species. The identity scores corresponding to half the population of *S. hermonthica *unigenes were 0.68 for grape and poplar, 0.65 for *Arabidopsis*, 0.62 for rice, and 0.56 for *P. patens*. These numbers roughly reflect the evolutionary distances between *S. hermonthica *and these species.

Large scale EST sequence datasets have previously been reported for *Triphysaria versicolor *[8] and *Triphysaria pusilla *[27], which are hemiparasitic plants belonging to the Orobanchaceae. The assembled EST sequences are available at the plantGDB web site [28]. Althoughthe genus *Triphysaria *is closely related taxonomically to *S. hermonthica*, only 74% of the *S. hermonthica *unigenes showed similarity to *Triphysaria *sequences (including both *T. pusilla *and *T. versicolor*), when analyzed with the tblastx program (Table 4). This is significantly lower than percentages of similarity found with the other dicotyledonous plants, but this is likely due to the lack of saturation of the *Triphysaria *EST datasets.

**Table 4:** Summary of blast search results using *S. hermonthica *unigenes.

Species	DB version	Number of hits	% Unigenes
*Populus trichocarpa*	JGI ver1.1	13,573	79.2
*Glycine max*	JGI ver1.1	12,716	79.0
*Vitis vinifera*	ver1	13,345	77.9
*Arabidopsis thaliana*	TAIR8	13,255	77.3
*Oryza sativa*	TIGR ver6	12,841	74.9
*Sorghum bicolor*	JGI ver1.1	12,803	74.7
*Triphysaria pusilla*	EST	12,716	74.2
*Physcomitrella patens*	JGI ver1.1	11,140	65.0
*Chlamydomonas reinhardtii*	JGI ver1.1	6,477	37.8
No hit		2,389	13.9

**Figure 4:**
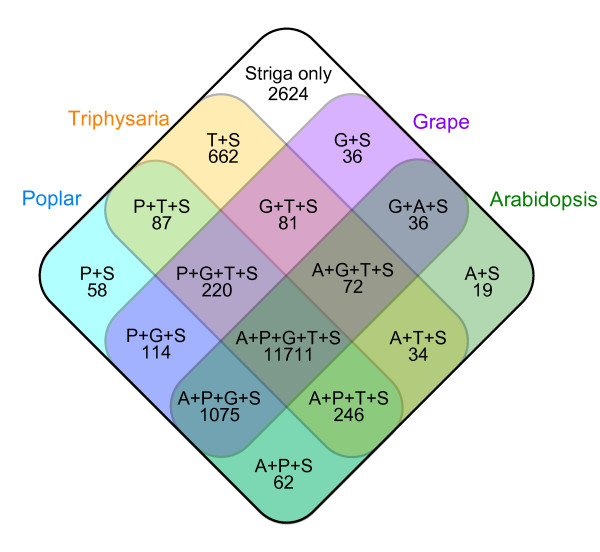
**Homologous gene groups between *S. hermonthica *and four other plant species**. The numbers of *S. hermonthica *unigenes that have homologues in the indicated plant species are represented by a Venn diagram. A: *A. thaliana*, G: *V. vinifera*, P: *P. trichocarpa*, T: *T. pusilla*or *T. versicolor*, and S: *S. hermonthica*.

The conservation of the genes between *S. hermonthica *and *Arabidopsis*, grape, poplar, or *Triphysaria *spp. is shown in a Venn diagram (Fig. 4). Among the 17,137 unigenes, 11,711 (68%) are conserved among all five groups. Only 19, 36, and 58 of the *S. hermonthica *unigenes are conserved specifically in *Arabidopsis*, grape, and poplar, respectively. Interestingly, we found that 662 (3.9%) of the *S. hermonthica *unigenes are conserved in *Triphysaria *spp. but not in *Arabidopsis*, grape, or poplar.

Of these 662 sequences, 73 show similarities to sequences in other databases such as rice, sorghum, soybean, *Physcomitrella*, UniRef90 or nr (the non-redundant peptide database from NCBI). We found no other homologs for the remaining 589 unigenes (Additional file 2). Since *T. pusilla *and *T. versicolor *are hemiparasitic plants, these 589 might include genes specific to parasitic plants. The ongoing project to sequence the genome of *Mimulus *spp. may help to narrow down the number of candidate genes that are involved in parasitism, because *Mimulus *spp. are non-parasitic members of the family Scrophulariaceae, which is taxonomically close to Orobanchaceae. The 2,389 unigenes (14%) that did not show significant hits with any known peptide sequences in the tested databases (including nr) are also listed in Additional file 2. These unigenes may include sequences that are specific to *Striga*.

### Genetic diversity of the *S. hermonthica *sequences

*S. hermonthica *is an obligate outcrossing plant with high levels of morphological and genetic variation [29]. The EST2uni program detected 9,299 putative single nucleotide polymorphisms (SNPs) among the *S. hermonthica *unigenes. To exclude the misidentification of sequencing errors as SNPs, only polymorphisms confirmed by at least 2 independent sequences were counted, although there is still the possibility that those polymorphisms occurred during cDNA synthesis. The average frequency of SNPs in the unigene sequences is 0.67%, or approximately 1 SNP per 1.5 kbp. Although these SNPs will need to be confirmed, these data will be useful for developing EST-SNP markers for *S. hermonthica *[30].

We found 1,445 di-, tri- or tetra-nucleotide microsatellites (or SSRs) among the *S. hermonthica *unigenes. The most frequent of these are the tri-nucleotide repeats (Additional file 3), which is in agreement with previous studies of other plant species [31-33]. The most frequent individual microsatellite repeat is AG (including TC, GA, and TC) (283, 19.6%) and the second most frequent is AC (including TG, CA, and GT) (218, 15.1%). The most frequent tri-nucleotide repeat is ATC (including TCA and CAT) (157, 11.0%) (Additional file 4).

The EST-SSR sequences are good candidates for genetic markers, which can be used for molecular diagnosis, for biotyping weeds, and for investigating the genetic diversity and population structures of *S. hermonthica*. To investigate whether the SSRs that we identified can be used as such markers, we designed primers using sequences flanking the putative SSRs and looked for polymorphisms by PCR. First, we pooled DNA samples extracted from the leaves of several plants in the same field and used the DNA pools as PCR templates. Of the 64 primer sets tested, 44 successfully amplified DNA bands. However, 26 primer sets (59%) produced smears or multiple bands that were not countable and only 18 primer pairs (41%) amplified clear separate bands (Additional file 5). The smeared bands may indicate heterozygosity and genetic diversity among the individual plants harvested from the same field. Therefore, we tested the individual plants for polymorphisms. Several markers that showed smear patterns from the pooled DNA templates actually amplified clear polymorphic bands from individual plants in the same population (Additional file 6). These data verify that *S. hermonthica *is a highly adaptable weed that has maintained a high degree of genetic variation and plasticity, to survive in various ecosystems [34].

### Genetic distances among *S. hermonthica *populations with different hosts

Although individual *S. hermonthica *plants possess highly diversified genomes, 18 of the primer sets we tested showed countable band patterns when using pooled DNA templates. Using those primer sets, we investigated the relationships between different *S. hermonthica *populations from 6 fields growing sorghum, maize, or pearl millet in various locations in Sudan or Kenya [35]. Of the 18 primer sets, 10 showed clear polymorphisms for different *S. hermonthica *populations (Table 5, Additional file 5). The analysis of PCR products was carried out using MultiNa^® ^(Shimadzu, Japan), a microchip electrophoresis system that permits the separation of small fragments and that can detect 5 bp differences. The average polymorphism information content (PIC) was 0.463, which confirms that the SSR markers used in this study were highly informative The lowest PIC value was 0.305 for SSR57, and the highest was 0.545 for SSR26 (Table 5). The analyzed loci included 3 di-, 3 tri-, and 4 tetra-nucleotide repeats. A total of 27 alleles were detected, with an average number of alleles per locus of 2.7. The genetic diversity among the six populations was revealed by the gene diversity values, which ranged from 0.375 to 0.625, with an average of 0.549. These results suggest a high level of diversity among the surveyed populations, as was expected for this obligate outcrossing plant [36-38].

**Table 5:** Genetic diversity among *S. hermonthica *populations collected from various locations and host plants.

SSR ID	Primer name	Repeat unit	No of repeats	No of alleles	Gene diversity	PIC
ShSSR_ShContig8678_1	SSR17	AC	18	3	0.611	0.535
ShSSR_ShContig6892_1	SSR26	AG	15	3	0.625	0.545
ShSSR_ShSHAA-aai51d05.b1_c_s_1	SSR33	AG	13	3	0.611	0.535
ShSSR_ShContig9253_1	SSR43	CCG	10	2	0.486	0.368
ShSSR_ShContig5481_1	SSR50	AAG	9	3	0.569	0.477
ShSSR_ShContig5198_1	SSR53	ACC	8	2	0.486	0.368
ShSSR_ShContig5533_1	SSR57	AACT	6	2	0.375	0.305
ShSSR_ShContig10128_1	SSR58	AAAC	7	3	0.569	0.505
ShSSR_ShSHAA-aab89e01.b1_c_s_1	SSR59	AAAC	6	3	0.542	0.460
ShSSR_ShContig9110_1	SSR63	AAAG	5	3	0.611	0.535
Average				2.700	0.549	0.463

**Figure 5:**
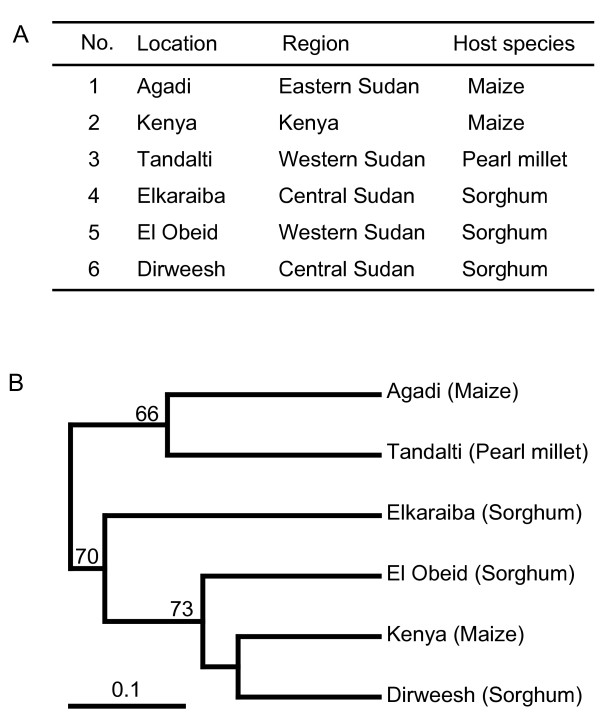
**Clustering analysis of *S. hermonthica* populations using SSR polymorphisms**. A. *S. hermonthica *populations used in this study. B. A UPGMA dendrogram constructed using polymorphisms at 10 SSR loci with a total of 27 alleles. Bootstrap values are indicated at supporting nodes when the values are greater than 50.

We also looked for correlations between host species and *S. hermonthica *biotypes, using the Unweighted Pair Group Method with Arithmetic mean (UPGMA) clustering analysis. The populations from El Obeid (host: sorghum), Dirweesh (host: sorghum), and Kenya (host: maize) clustered in one group, while the population from Elkaraiba (host: sorghum) was in a distant branch of the same group. Those from Tandalti (host: pearl millet) and Agadi (host: maize) formed another cluster (Fig. 5). Thus, we did not detect any correlations between genetic distance and host specificity in this study. This result is consistent with previous epidemiological reports [35,38-40]. In summary, our results suggest that the SSRs found in our study could be useful tools for further investigations of genetic diversity in *S. hermonthica*.

### Web-based database

The results of the sequencing and analysis of the *S. hermonthica *ESTs are freely available online from our web-based database [9]. The web interface was based on the original EST2uni web site [15]. The database contains features for complex query searches and a blast search. A page for each unigene consists of its sequence, contig images, results of blast similarity searches, lists of detected SSRs and SNPs, and GO categorizations. In addition, the homologs of each unigene are linked to outside databases such as The Arabidopsis Information Resource (TAIR) [41]. This web-based database will be a powerful tool for the detailed analysis of *S. hermonthica *genes.

## Conclusions

This paper provides large scale EST information about *S. hermonthica*, which can be used in studies of parasitic plants, plant-plant interactions, weed management, and plant evolution. Comparative analyses between *S. hermonthica *and other plant genomes should allow us to identify genes responsible for plant parasitism. These genes are of particular interest as potential targets for future pest management strategies against noxious parasitic weeds. Our analysis also highlights the intra-species genetic diversity of *S. hermonthica*. A more detailed analysis might contribute to future breeding programs to develop resistant crops, since genetic variation in the weed population could be the main factor allowing the quick breakdown of resistance. In summary, our study provides powerful analytical tools for the molecular analysis of the parasitic weed *S. hermonthica*. Our data will alsocontribute to the annotation of genes identified by the on-going genome-scale sequencing of the parasitic genera from Orobanchaceae.

## Methods

### Plant materials and growth conditions

*S. hermonthica *seeds collected from a sorghum field in 1994 in Kenya were provided by Dr. A. G. Babiker (Univ. of Sudan, Khartoum, Sudan). Rice seeds (*Oryza sativa *L. subspecies *japonica*, cultivar Koshihikari) were originally obtained from the National Institute of Agricultural Sciences (NIAS, Tsukuba, Japan). *S. hermonthica *plants parasitizing rice were grown in rhizotrons as described previously [42] or in soil (1:1 mixture of vermiculite: clay). For the axenic culture of *S. hermonthica*, seeds were sterilized with 20% bleach solution (approx. 6% NaOCl) for 5 min and washed thoroughly with sterile water. The sterile seeds were preconditioned on MS medium with 1% sucrose and 0.5% phytagel (Sigma) at 26°C for 7 to 10 days in the dark and germination was stimulated by the exogenous application of 5 μl 1 μM Strigol per plate. Sterile *S. hermonthica *plants were grown on the same medium at 26°C with a 16-h photoperiod, and the medium was renewed every 3 weeks.

### Determination of nuclear DNA content

The nuclear DNA content was analyzed with a flow cytometer (Partec PA, Tokyo, Japan). Soil-grown *S. hermonthica *(host: rice) leaves were chopped with a razor blade into small pieces and analyzed according to the previously published method [43]. Leaves of *Arabidopsis *(ecotype Col -0) were used as the control.

### RNA extraction

The *S. hermonthica *tissues and developmental stages used for RNA extraction are listed in Table 1. *S. hermonthica *RNAs were extracted using a modified cetyl trimethylammonium bromide (CTAB) method. Briefly, plant tissues were ground under liquid nitrogen and suspended in 5 × volumes of CTAB solution (2% CTAB, 2% polybinylpyrrolidone (PVP), 25 mM ethylenediaminetetraacetic acid(EDTA), 2 M NaCl, 1% beta-mercaptoethanol, 100 mM Tris-HCl (pH 8.0)) and phenol:chloroform (5:1, pH 4.7, Sigma). The mixtures were shaken at 55°C for 5 min. After 10 min centrifugation, the aqueous phase was extracted with an equal volume of phenol:chloroform, and subsequently with chloroform. The RNAs were precipitated by adding 0.25 volumes of 10 M LiCl. The RNA pellet was washed with 70% ethanol and then dissolved in nuclease-free water. Samples were subsequently purified using the PureLink RNA mini kit (Invitrogen) according to the manufacture's instructions. To obtain mRNA for library construction, total RNAs from each tissue and developmental stage were mixed and purified using an mRNA purification kit (GE) according to the manufacture's instructions. The quality and quantity of the total RNA and the mRNA were assessed by measurements of OD_230_, OD_260_, and OD_280_, followed by visual checking by electrophoresis.

### Library construction and EST sequencing

The construction of the normalized, full-length enriched library was carried out in Evrogen (Russia). The cDNA normalization was conducted using a Duplex-specific nuclease (DSN)-based method, and full-length cDNAs were enriched using the SMART™ technology (Clontech). Each cDNA was inserted into the pAL17.3 vector. Sequencing of randomly picked clones was performed in the Genome Center at Washington University using the ABI3730 capillary sequencer.

### Computational analysis

The EST sequences were automatically trimmed, clustered and annotated using the EST2uni analysis pipeline [15]. Sequence assembly was performed using the CAP3 program with the default parameter settings [44]. Blast searches were performed with NCBI blast program against the databases shown in Table 4. The *S. hermonthica *online database was constructed based on the EST2uni web program with slight modifications.

### SSR markers and genetic diversity analysis

Genomic DNA was extracted from about 10 g of *S. hermonthica *seeds using the modified CTAB method described previously [35]. Primers flanking the microsatellites were designed using the PRIMER 3 program [45]. The PCRs were performed in 10 μl volumes with one initial denaturation step of 1 min at 95°C, followed by 40 cycles of 15 sec at 94°C, 30 sec at 60°C and 30 sec at 72°C, anda final extension step of 5 min at 72°C. The PCR products were analyzed either by 4% agarose gel electrophoresis (Additional file 6) or using the MCE-202 MultiNa Microchip Electrophoresis System for DNA/RNA analysis (Shimadzu, Japan) using the DNA-500 kit (Table 5 and Fig. 5). The data were analyzedusing the PowerMarker program version 3.25 [46], and the genetic diversity was estimated based on allelic numbersand the gene diversity value:

where *n *is the number of populations sampled, *p*_*lu *_is the frequency of *u*th allele at the *l*th locus, and *f *is the inbreeding coefficient (association between alleles) at the *l*th locus. The Polymorphism Information Content (PIC) was estimated as , where the p_*lv *_is the frequency of the *v*th allele at the *l*th locus. The phylogenetic UPGMA tree was generated based on a matrix of the frequencies and distances using the LogSharedAllele algorithm with the PowerMarker v.3.25 program. Bootstrap analysis was performed using the software package WINBOOT [47].

## Authors' contributions

SY carried out the data collection and bioinformatic analyses, and drafted the manuscript. JKI performed the SSR marker analyses. NMK and AMA collected *S. hermonthica *seeds and extracted genomic DNAs. SN participated in the design and coordination of the study. KS conceived of the study, contributed to designing the experiments, and drafted the manuscript. All authors read and approved the final manuscript.

## Supplementary Material

Additional file 1 **Distribution of unigene lengths and EST numbers per unigene**. (A) Distribution of unigene lengths in the entire *S. hermonthica *unigene dataset. (B) Distribution of EST numbers per unigene. Click here for file

Additional file 2 **Lists of *S. hermonthica *unigenes that are potentially specific to parasitic plants**. Sheet 1- The list of *S. hermonthica *unigenes that have homologs in *T. pusilla *or *T. versicolor *but not in other species databases. Sheet 2- The list of *S. hermonthica *unigenes that do not have homologs in other known sequences. Click here for file

Additional file 3 Distribution of SSR patterns detected in *S. hermonthica *ESTs. Click here for file

Additional file 4 Distribution of SSR motifs detected in S. hermonthica ESTs. Click here for file

Additional file 5 **SSR information**. Sheet1- The list of SSRs analyzed in this study, with SSR ID, primer sequences, and PCR results. The yellow colored linesindicate the markers used in this study. Click here for file

Additional file 6 **Examples of PCR results from the amplification of SSR-containing regions in *S. hermonthica***. (A) Agarose gel images of PCR results using the indicated primer sets and pooled genomic DNAs from the populations listed in Fig. 5. The population numbers correspond to the numbers in Fig. 5A. (B) An agarose gel image showing PCR results using the SSR8 primer set and genomic DNAs extracted from individual plantsfrom the population in Kenya. Click here for file
