# Supplementary figures and images for: A full-length enriched cDNA library and expressed sequence tag analysis of the parasitic weed, Striga hermonthica
Source: BMC Plant Biol. 2010 Mar 30;10:55. doi: 10.1186/1471-2229-10-55 (PMC2923529; doi:10.1186/1471-2229-10-55)

**A** Length distribution

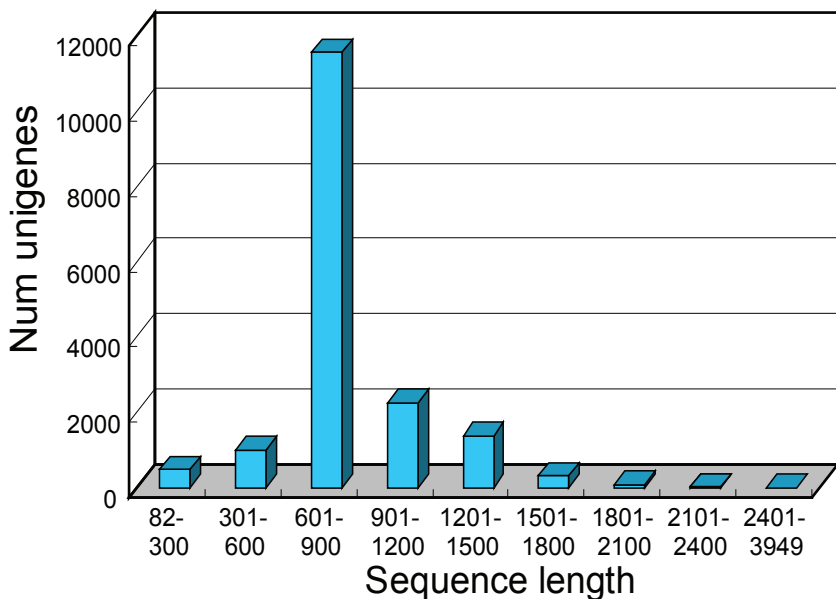

**B** EST number per unigene distribution

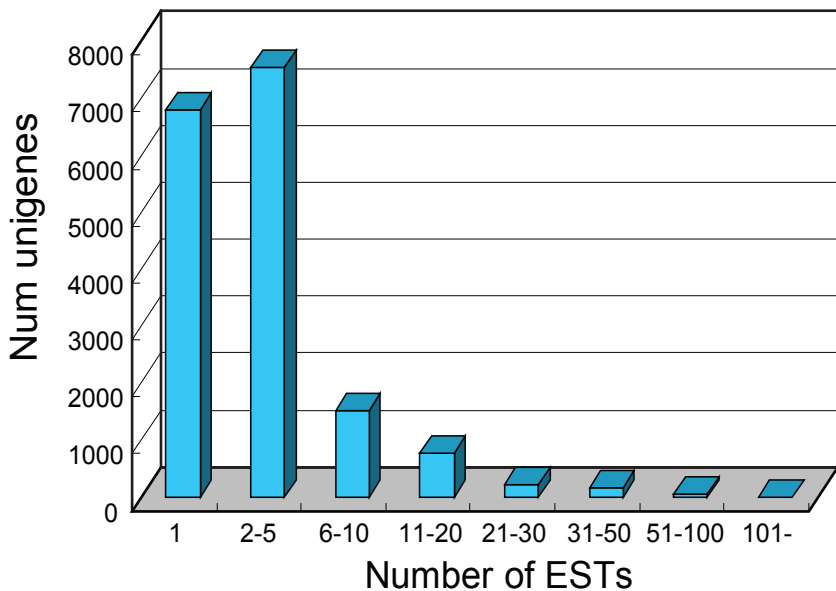

Supplement: Additional file 1 — Distribution of unigene lengths and EST numbers per unigene. (A) Distribution of unigene lengths in the entire S. hermonthica unigene dataset. (B) Distribution of EST numbers per unigene. [file 1471-2229-10-55-S1.PDF]

A

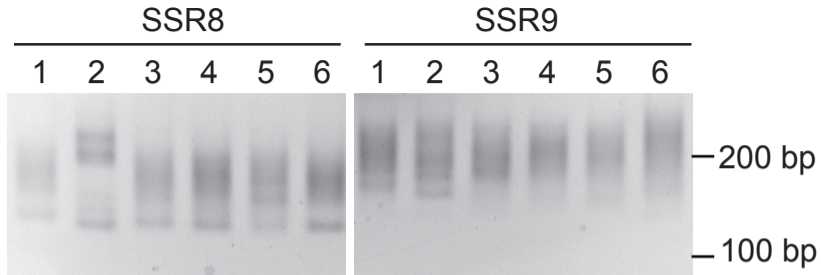

B

SSR8 (individual plants)

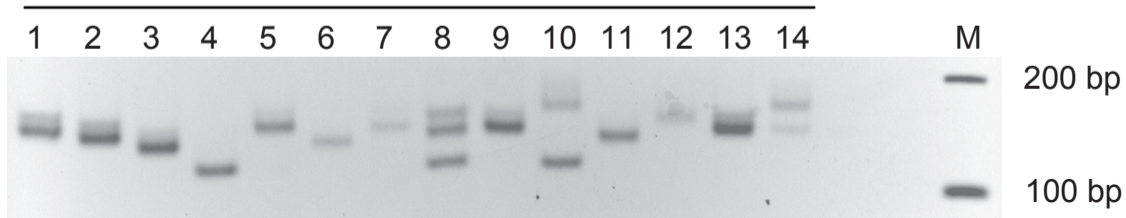

Supplement: Additional file 6 — Examples of PCR results from the amplification of SSR-containing regions in S. hermonthica. (A) Agarose gel images of PCR results using the indicated primer sets and pooled genomic DNAs from the populations listed in Fig. 5. The population numbers correspond to the numbers in Fig. 5A. (B) An agarose gel image showing PCR results using the SSR8 primer set and genomic DNAs extracted from individual plantsfrom the population in Kenya. [file 1471-2229-10-55-S6.PDF]
