# Supplementary material for: A full-length enriched cDNA library and expressed sequence tag analysis of the parasitic weed, Striga hermonthica
Source: BMC Plant Biol. 2010 Mar 30;10:55. doi: 10.1186/1471-2229-10-55 (PMC2923529; doi:10.1186/1471-2229-10-55)
Supplement: Additional file 3 — Distribution of SSR patterns detected in S. hermonthica ESTs. [file 1471-2229-10-55-S3.PDF]

Additional file 3 - Distribution of SSR patterns detected in *S. hermonthica* ESTs.

Pattern	number	%
dinucleotide repeat	536	37.1
trinucleotide repeat	673	46.6
tetranucleotide repeat	236	16.3
Total	1,445	
