# Supplementary material for: A full-length enriched cDNA library and expressed sequence tag analysis of the parasitic weed, Striga hermonthica
Source: BMC Plant Biol. 2010 Mar 30;10:55. doi: 10.1186/1471-2229-10-55 (PMC2923529; doi:10.1186/1471-2229-10-55)
Supplement: Additional file 4 — Distribution of SSR motifs detected in S. hermonthica ESTs. [file 1471-2229-10-55-S4.PDF]

Additional file 4- Distribution of SSR motifs detected in *S. hermonthica* ESTs.

Unit	Number of pSSR
AG/CT	283
AC/GT	218
ATC/GAT	157
CCG/CGG	120
AAG/CTT	118
AAAT/ATTT	107
AAT/TTA	93
AGC/GCT	52
ACC/GGT	42
AAC/GTT	38
AAAC/GTTT	37
AGG/CCT	37
AT/TA	34
AAAG/CTTT	23
ACG/CGT	15
AATC/GATT	9
AGAT/ATCT	9
AATT/AATT	8
ACTC/GAGT	6
ACAT/ATGT	5
AACC/GGTT	4
AAGG/CCTT	4
AGCG/CGCT	3
AACG/CGTT	3
ATCC/GGAT	3
AATG/CATT	3
ATCG/CGAT	2
CCGG/CCGG	2
ACGC/GCGT	2
ACCG/CGGT	2
AACT/AGTT	2
AGCT/AGCT	1
ACT/AGT	1
ACAG/CTGT	1
CG/CG	1
Total	1445
